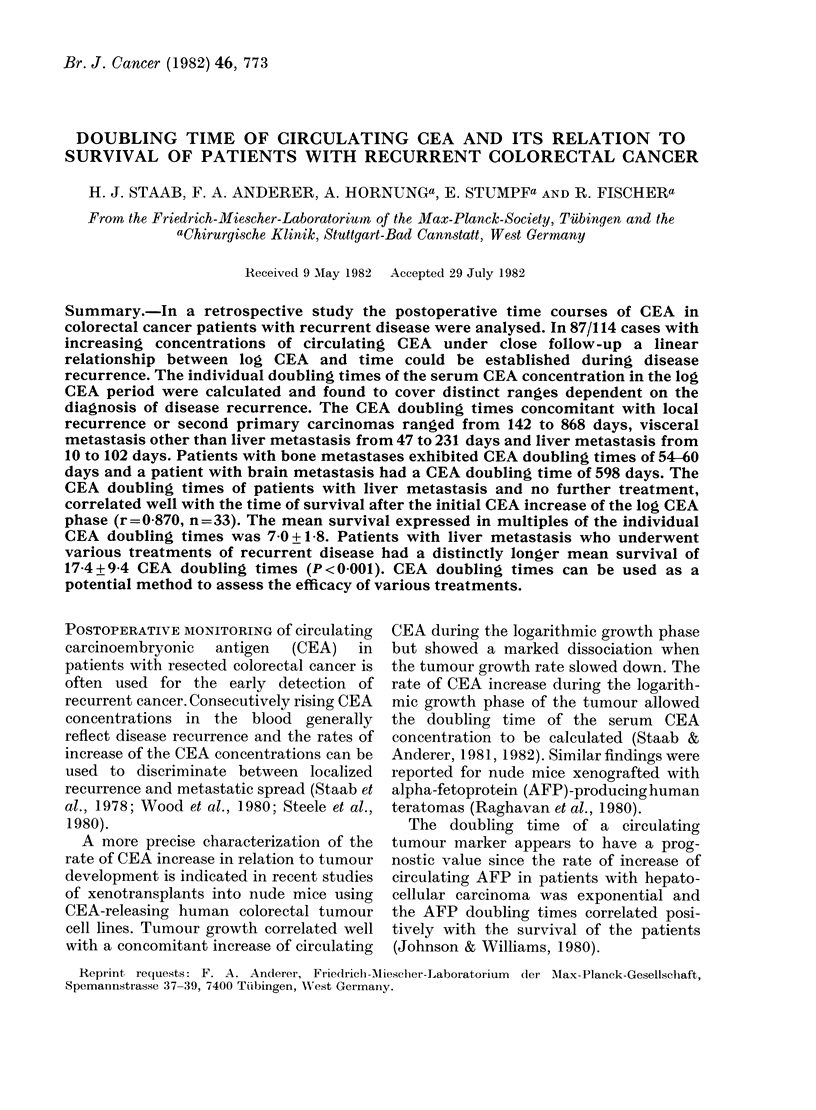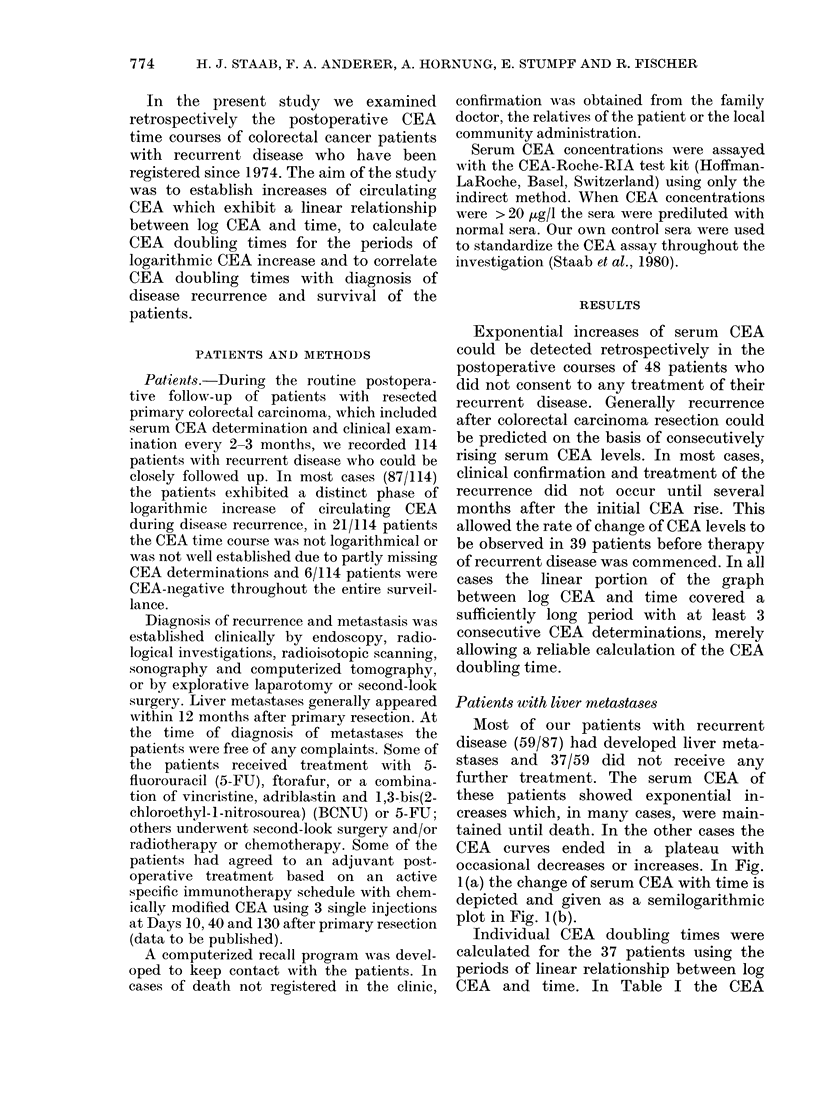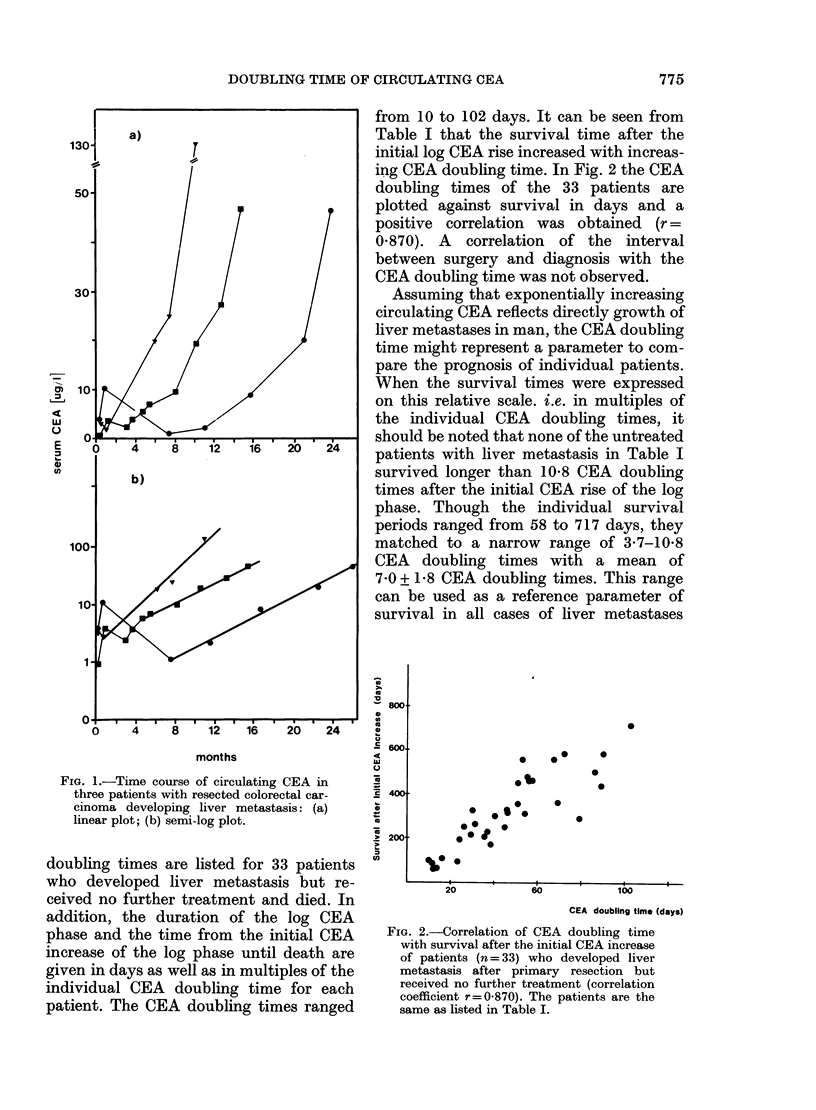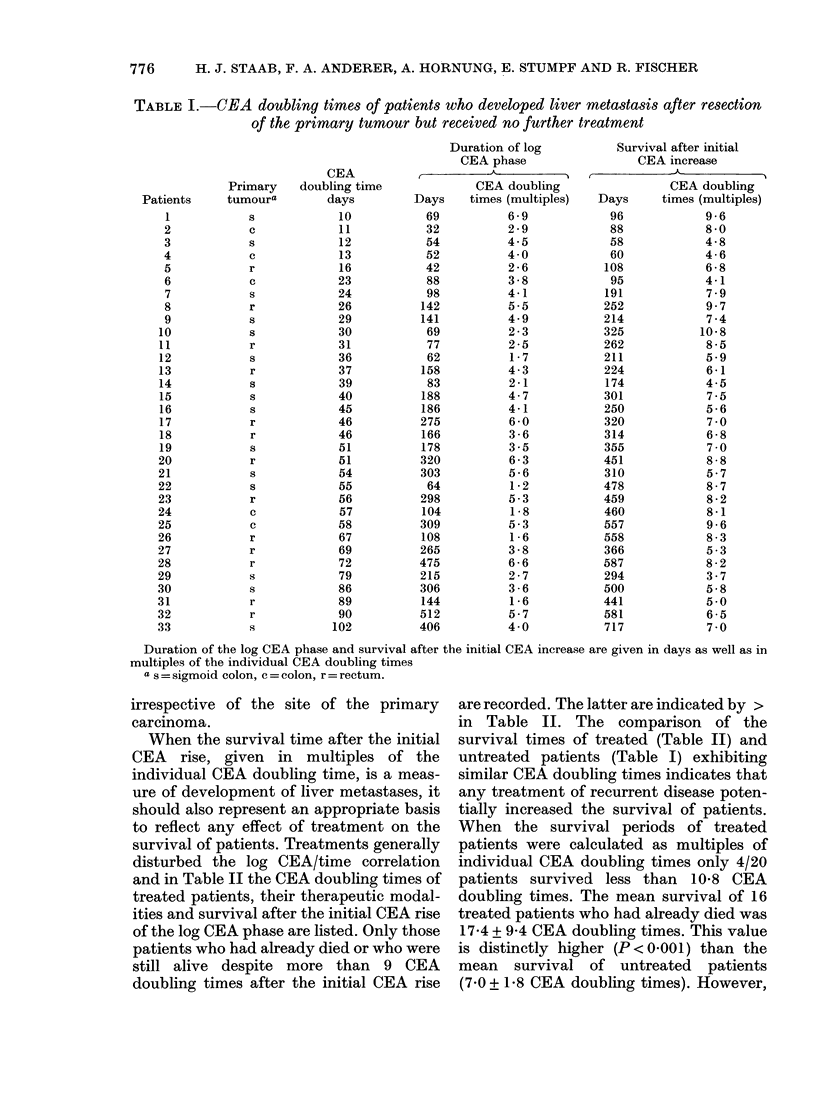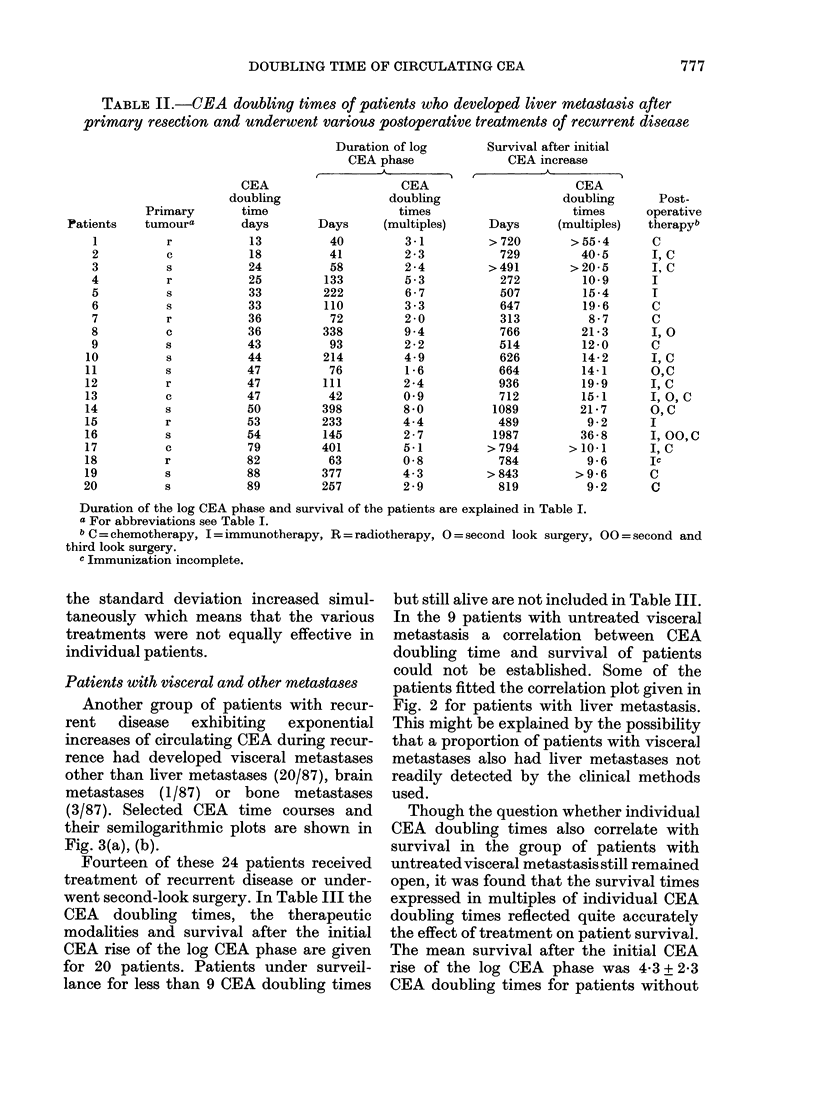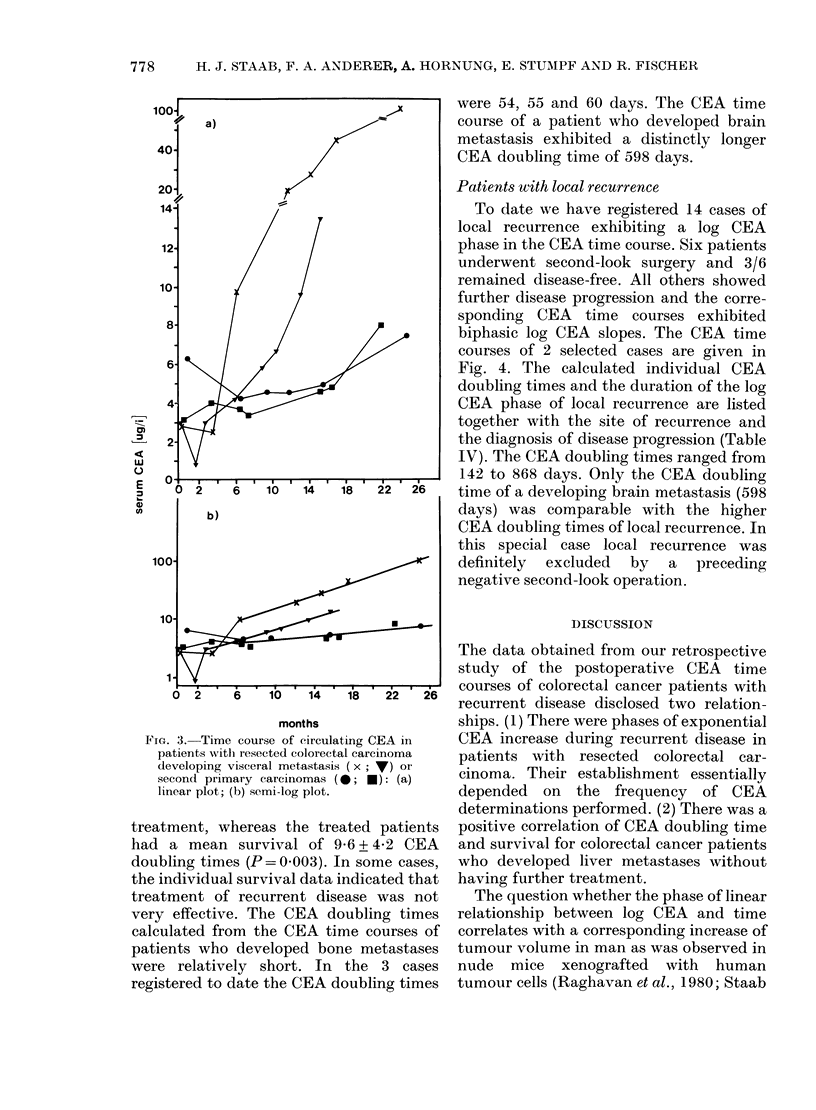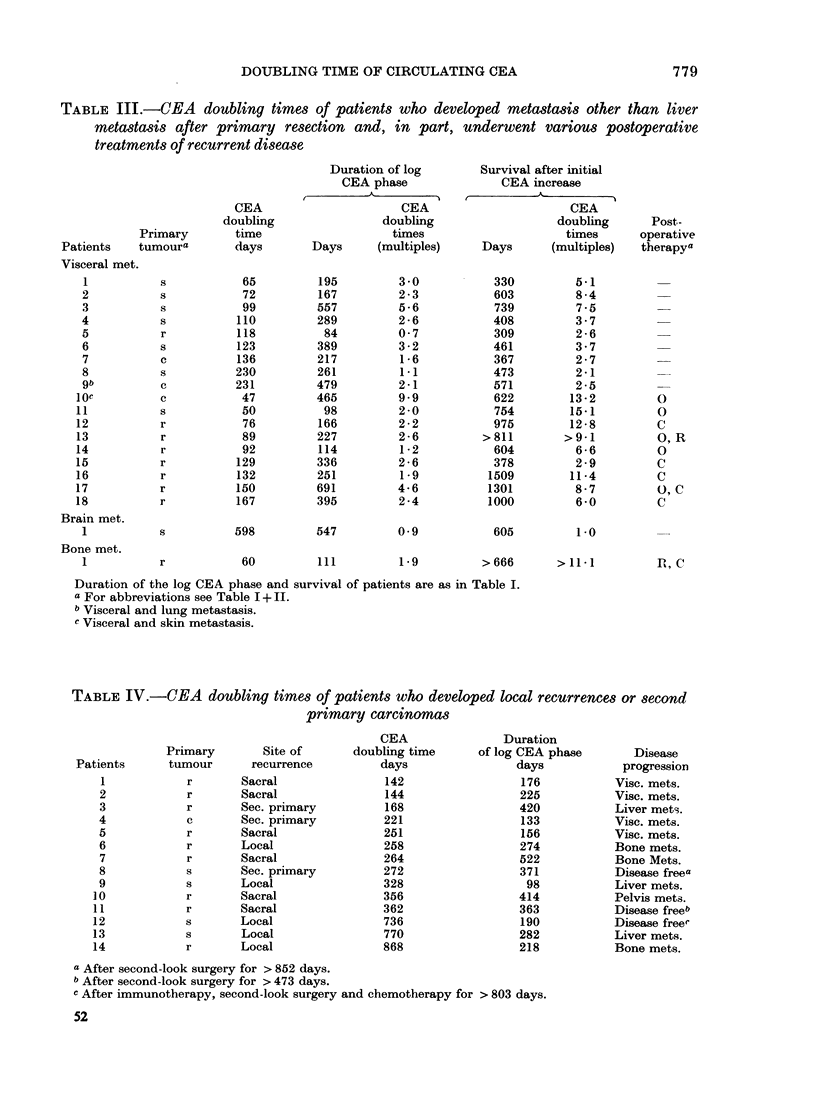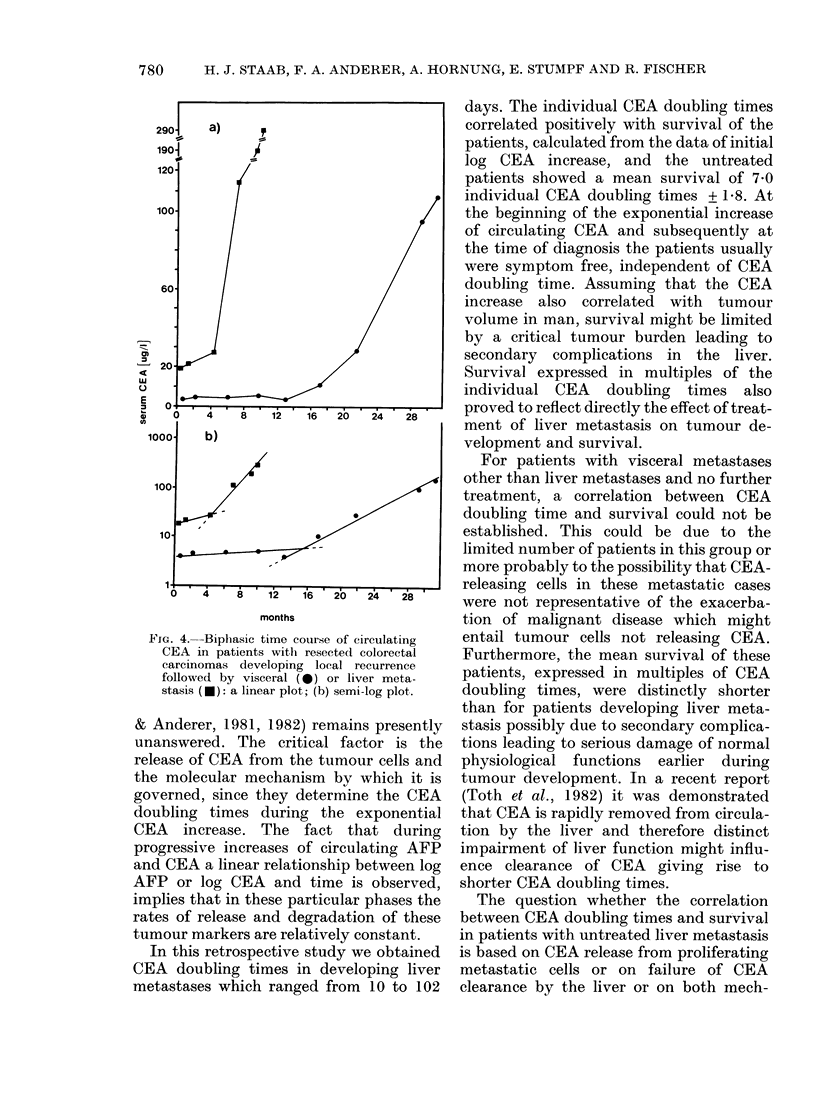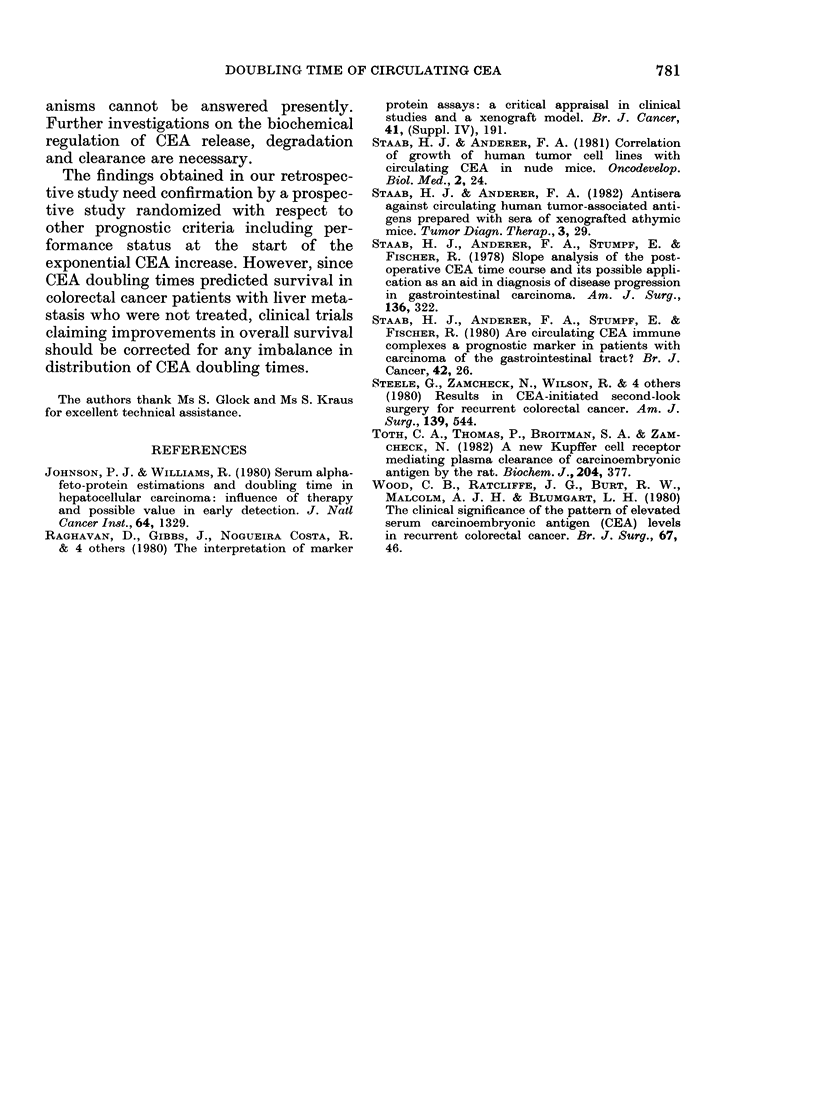# Doubling time of circulating cea and its relation to survival of patients with recurrent colorectal cancer.

**DOI:** 10.1038/bjc.1982.270

**Published:** 1982-11

**Authors:** H. J. Staab, F. A. Anderer, A. Hornung, E. Stumpf, R. Fischer

## Abstract

In a retrospective study the postoperative time courses of CEA in colorectal cancer patients with recurrent disease were analysed. In 87/114 cases with increasing concentrations of circulating CEA under close follow-up a linear relationship between log CEA and time could be established during disease recurrence. The individual doubling times of the serum CEA concentration in the log CEA period were calculated and found to cover distinct ranges dependent on the diagnosis of disease recurrence. The CEA doubling times concomitant with local recurrence or second primary carcinomas ranged from 142 to 868 days, visceral metastasis other than liver metastasis from 47 to 231 days and liver metastasis from 10 to 102 days. Patients with bone metastases exhibited CEA doubling times of 54-60 days and a patient with brain metastasis had a CEA doubling time of 598 days. The CEA doubling times of patients with liver metastasis and no further treatment, correlated well with the time of survival after the initial CEA increase of the log CEA phase (r = 0.870, n = 33). The mean survival expressed in multiples of the individual CEA doubling times was 7.0 +/- 1.8. Patients with liver metastasis who underwent various treatments of recurrent disease had a distinctly longer mean survival of 17.4 +/- 9.4 CEA doubling times (P less than 0.001). CEA doubling times can be used as a potential method to assess the efficacy of various treatments.


*Br. J. Cancer* (1982) **46**, 773

### DOUBLING TIME OF CIRCULATING CEA AND ITS RELATION TO SURVIVAL OF PATIENTS WITH RECURRENT COLORECTAL CANCER

H. J. STAAB, F. A. ANDERER, A. HORNUNG[a], E. STUMPF[a] AND R. FISCHER[a]

*From the Friedrich-Miescher-Laboratorium of the Max-Planck-Society, Tübingen and the [a]Chirurgische Klinik, Stuttgart-Bad Cannstatt, West Germany*

Received 9 May 1982 Accepted 29 July 1982

**Summary.—In a retrospective study the postoperative time courses of CEA in colorectal cancer patients with recurrent disease were analysed. In 87/114 cases with increasing concentrations of circulating CEA under close follow-up a linear relationship between log CEA and time could be established during disease recurrence. The individual doubling times of the serum CEA concentration in the log CEA period were calculated and found to cover distinct ranges dependent on the diagnosis of disease recurrence. The CEA doubling times concomitant with local recurrence or second primary carcinomas ranged from 142 to 868 days, visceral metastasis other than liver metastasis from 47 to 231 days and liver metastasis from 10 to 102 days. Patients with bone metastases exhibited CEA doubling times of 54–60 days and a patient with brain metastasis had a CEA doubling time of 598 days. The CEA doubling times of patients with liver metastasis and no further treatment, correlated well with the time of survival after the initial CEA increase of the log CEA phase ($r=0{\cdot}870$, $n=33$). The mean survival expressed in multiples of the individual CEA doubling times was $7{\cdot}0 \pm 1{\cdot}8$. Patients with liver metastasis who underwent various treatments of recurrent disease had a distinctly longer mean survival of $17{\cdot}4 \pm 9{\cdot}4$ CEA doubling times ($P<0{\cdot}001$). CEA doubling times can be used as a potential method to assess the efficacy of various treatments.**

POSTOPERATIVE MONITORING of circulating carcinoembryonic antigen (CEA) in patients with resected colorectal cancer is often used for the early detection of recurrent cancer. Consecutively rising CEA concentrations in the blood generally reflect disease recurrence and the rates of increase of the CEA concentrations can be used to discriminate between localized recurrence and metastatic spread (Staab *et al.*, 1978; Wood *et al.*, 1980; Steele *et al.*, 1980).

A more precise characterization of the rate of CEA increase in relation to tumour development is indicated in recent studies of xenotransplants into nude mice using CEA-releasing human colorectal tumour cell lines. Tumour growth correlated well with a concomitant increase of circulating CEA during the logarithmic growth phase but showed a marked dissociation when the tumour growth rate slowed down. The rate of CEA increase during the logarithmic growth phase of the tumour allowed the doubling time of the serum CEA concentration to be calculated (Staab & Anderer, 1981, 1982). Similar findings were reported for nude mice xenografted with alpha-fetoprotein (AFP)-producing human teratomas (Raghavan *et al.*, 1980).

The doubling time of a circulating tumour marker appears to have a prognostic value since the rate of increase of circulating AFP in patients with hepatocellular carcinoma was exponential and the AFP doubling times correlated positively with the survival of the patients (Johnson & Williams, 1980).

Reprint requests: F. A. Anderer, Friedrich-Miescher-Laboratorium der Max-Planck-Gesellschaft, Spemannstrasse 37–39, 7400 Tübingen, West Germany.

In the present study we examined retrospectively the postoperative CEA time courses of colorectal cancer patients with recurrent disease who have been registered since 1974. The aim of the study was to establish increases of circulating CEA which exhibit a linear relationship between log CEA and time, to calculate CEA doubling times for the periods of logarithmic CEA increase and to correlate CEA doubling times with diagnosis of disease recurrence and survival of the patients.

### PATIENTS AND METHODS

*Patients.*—During the routine postoperative follow-up of patients with resected primary colorectal carcinoma, which included serum CEA determination and clinical examination every 2–3 months, we recorded 114 patients with recurrent disease who could be closely followed up. In most cases (87/114) the patients exhibited a distinct phase of logarithmic increase of circulating CEA during disease recurrence, in 21/114 patients the CEA time course was not logarithmical or was not well established due to partly missing CEA determinations and 6/114 patients were CEA-negative throughout the entire surveillance.

Diagnosis of recurrence and metastasis was established clinically by endoscopy, radiological investigations, radioisotopic scanning, sonography and computerized tomography, or by explorative laparotomy or second-look surgery. Liver metastases generally appeared within 12 months after primary resection. At the time of diagnosis of metastases the patients were free of any complaints. Some of the patients received treatment with 5-fluorouracil (5-FU), ftorafur, or a combination of vincristine, adriblastin and 1,3-bis(2-chloroethyl-1-nitrosourea) (BCNU) or 5-FU; others underwent second-look surgery and/or radiotherapy or chemotherapy. Some of the patients had agreed to an adjuvant postoperative treatment based on an active specific immunotherapy schedule with chemically modified CEA using 3 single injections at Days 10, 40 and 130 after primary resection (data to be published).

A computerized recall program was developed to keep contact with the patients. In cases of death not registered in the clinic, confirmation was obtained from the family doctor, the relatives of the patient or the local community administration.

Serum CEA concentrations were assayed with the CEA-Roche-RIA test kit (Hoffman-LaRoche, Basel, Switzerland) using only the indirect method. When CEA concentrations were $>20$ $\mu g/l$ the sera were prediluted with normal sera. Our own control sera were used to standardize the CEA assay throughout the investigation (Staab *et al.*, 1980).

### RESULTS

Exponential increases of serum CEA could be detected retrospectively in the postoperative courses of 48 patients who did not consent to any treatment of their recurrent disease. Generally recurrence after colorectal carcinoma resection could be predicted on the basis of consecutively rising serum CEA levels. In most cases, clinical confirmation and treatment of the recurrence did not occur until several months after the initial CEA rise. This allowed the rate of change of CEA levels to be observed in 39 patients before therapy of recurrent disease was commenced. In all cases the linear portion of the graph between log CEA and time covered a sufficiently long period with at least 3 consecutive CEA determinations, merely allowing a reliable calculation of the CEA doubling time.

### *Patients with liver metastases*

Most of our patients with recurrent disease (59/87) had developed liver metastases and 37/59 did not receive any further treatment. The serum CEA of these patients showed exponential increases which, in many cases, were maintained until death. In the other cases the CEA curves ended in a plateau with occasional decreases or increases. In Fig. 1(a) the change of serum CEA with time is depicted and given as a semilogarithmic plot in Fig. 1(b).

Individual CEA doubling times were calculated for the 37 patients using the periods of linear relationship between log CEA and time. In Table I the CEA

FIG. 1.—Time course of circulating CEA in three patients with resected colorectal carcinoma developing liver metastasis: (a) linear plot; (b) semi-log plot.

doubling times are listed for 33 patients who developed liver metastasis but received no further treatment and died. In addition, the duration of the log CEA phase and the time from the initial CEA increase of the log phase until death are given in days as well as in multiples of the individual CEA doubling time for each patient. The CEA doubling times ranged from 10 to 102 days. It can be seen from Table I that the survival time after the initial log CEA rise increased with increasing CEA doubling time. In Fig. 2 the CEA doubling times of the 33 patients are plotted against survival in days and a positive correlation was obtained ($r = 0{\cdot}870$). A correlation of the interval between surgery and diagnosis with the CEA doubling time was not observed.

Assuming that exponentially increasing circulating CEA reflects directly growth of liver metastases in man, the CEA doubling time might represent a parameter to compare the prognosis of individual patients. When the survival times were expressed on this relative scale. *i.e.* in multiples of the individual CEA doubling times, it should be noted that none of the untreated patients with liver metastasis in Table I survived longer than 10·8 CEA doubling times after the initial CEA rise of the log phase. Though the individual survival periods ranged from 58 to 717 days, they matched to a narrow range of 3·7–10·8 CEA doubling times with a mean of $7{\cdot}0 \pm 1{\cdot}8$ CEA doubling times. This range can be used as a reference parameter of survival in all cases of liver metastases

FIG. 2.—Correlation of CEA doubling time with survival after the initial CEA increase of patients ($n = 33$) who developed liver metastasis after primary resection but received no further treatment (correlation coefficient $r = 0{\cdot}870$). The patients are the same as listed in Table I.

TABLE I.—*CEA doubling times of patients who developed liver metastasis after resection of the primary tumour but received no further treatment*

| Patients | Primary tumour[a] | CEA doubling time days | Duration of log CEA phase: Days | Duration of log CEA phase: CEA doubling times (multiples) | Survival after initial CEA increase: Days | Survival after initial CEA increase: CEA doubling times (multiples) |
|---|---|---|---|---|---|---|
| 1 | s | 10 | 69 | 6·9 | 96 | 9·6 |
| 2 | c | 11 | 32 | 2·9 | 88 | 8·0 |
| 3 | s | 12 | 54 | 4·5 | 58 | 4·8 |
| 4 | c | 13 | 52 | 4·0 | 60 | 4·6 |
| 5 | r | 16 | 42 | 2·6 | 108 | 6·8 |
| 6 | c | 23 | 88 | 3·8 | 95 | 4·1 |
| 7 | s | 24 | 98 | 4·1 | 191 | 7·9 |
| 8 | r | 26 | 142 | 5·5 | 252 | 9·7 |
| 9 | s | 29 | 141 | 4·9 | 214 | 7·4 |
| 10 | s | 30 | 69 | 2·3 | 325 | 10·8 |
| 11 | r | 31 | 77 | 2·5 | 262 | 8·5 |
| 12 | s | 36 | 62 | 1·7 | 211 | 5·9 |
| 13 | r | 37 | 158 | 4·3 | 224 | 6·1 |
| 14 | s | 39 | 83 | 2·1 | 174 | 4·5 |
| 15 | s | 40 | 188 | 4·7 | 301 | 7·5 |
| 16 | s | 45 | 186 | 4·1 | 250 | 5·6 |
| 17 | r | 46 | 275 | 6·0 | 320 | 7·0 |
| 18 | r | 46 | 166 | 3·6 | 314 | 6·8 |
| 19 | s | 51 | 178 | 3·5 | 355 | 7·0 |
| 20 | r | 51 | 320 | 6·3 | 451 | 8·8 |
| 21 | s | 54 | 303 | 5·6 | 310 | 5·7 |
| 22 | s | 55 | 64 | 1·2 | 478 | 8·7 |
| 23 | r | 56 | 298 | 5·3 | 459 | 8·2 |
| 24 | c | 57 | 104 | 1·8 | 460 | 8·1 |
| 25 | c | 58 | 309 | 5·3 | 557 | 9·6 |
| 26 | r | 67 | 108 | 1·6 | 558 | 8·3 |
| 27 | r | 69 | 265 | 3·8 | 366 | 5·3 |
| 28 | r | 72 | 475 | 6·6 | 587 | 8·2 |
| 29 | s | 79 | 215 | 2·7 | 294 | 3·7 |
| 30 | s | 86 | 306 | 3·6 | 500 | 5·8 |
| 31 | r | 89 | 144 | 1·6 | 441 | 5·0 |
| 32 | r | 90 | 512 | 5·7 | 581 | 6·5 |
| 33 | s | 102 | 406 | 4·0 | 717 | 7·0 |

Duration of the log CEA phase and survival after the initial CEA increase are given in days as well as in multiples of the individual CEA doubling times

[a] s = sigmoid colon, c = colon, r = rectum.

irrespective of the site of the primary carcinoma.

When the survival time after the initial CEA rise, given in multiples of the individual CEA doubling time, is a measure of development of liver metastases, it should also represent an appropriate basis to reflect any effect of treatment on the survival of patients. Treatments generally disturbed the log CEA/time correlation and in Table II the CEA doubling times of treated patients, their therapeutic modalities and survival after the initial CEA rise of the log CEA phase are listed. Only those patients who had already died or who were still alive despite more than 9 CEA doubling times after the initial CEA rise are recorded. The latter are indicated by > in Table II. The comparison of the survival times of treated (Table II) and untreated patients (Table I) exhibiting similar CEA doubling times indicates that any treatment of recurrent disease potentially increased the survival of patients. When the survival periods of treated patients were calculated as multiples of individual CEA doubling times only 4/20 patients survived less than 10·8 CEA doubling times. The mean survival of 16 treated patients who had already died was $17{\cdot}4 \pm 9{\cdot}4$ CEA doubling times. This value is distinctly higher ($P < 0{\cdot}001$) than the mean survival of untreated patients ($7{\cdot}0 \pm 1{\cdot}8$ CEA doubling times). However,

TABLE II.—*CEA doubling times of patients who developed liver metastasis after primary resection and underwent various postoperative treatments of recurrent disease*

| | | | Duration of log CEA phase | | Survival after initial CEA increase | | |
|---|---|---|---|---|---|---|---|
| Patients | Primary tumour[a] | CEA doubling time days | Days | CEA doubling times (multiples) | Days | CEA doubling times (multiples) | Post-operative therapy[b] |
| 1 | r | 13 | 40 | 3·1 | >720 | >55·4 | C |
| 2 | c | 18 | 41 | 2·3 | 729 | 40·5 | I, C |
| 3 | s | 24 | 58 | 2·4 | >491 | >20·5 | I, C |
| 4 | r | 25 | 133 | 5·3 | 272 | 10·9 | I |
| 5 | s | 33 | 222 | 6·7 | 507 | 15·4 | I |
| 6 | s | 33 | 110 | 3·3 | 647 | 19·6 | C |
| 7 | r | 36 | 72 | 2·0 | 313 | 8·7 | C |
| 8 | c | 36 | 338 | 9·4 | 766 | 21·3 | I, O |
| 9 | s | 43 | 93 | 2·2 | 514 | 12·0 | C |
| 10 | s | 44 | 214 | 4·9 | 626 | 14·2 | I, C |
| 11 | s | 47 | 76 | 1·6 | 664 | 14·1 | O,C |
| 12 | r | 47 | 111 | 2·4 | 936 | 19·9 | I, C |
| 13 | c | 47 | 42 | 0·9 | 712 | 15·1 | I, O, C |
| 14 | s | 50 | 398 | 8·0 | 1089 | 21·7 | O, C |
| 15 | r | 53 | 233 | 4·4 | 489 | 9·2 | I |
| 16 | s | 54 | 145 | 2·7 | 1987 | 36·8 | I, OO, C |
| 17 | c | 79 | 401 | 5·1 | >794 | >10·1 | I, C |
| 18 | r | 82 | 63 | 0·8 | 784 | 9·6 | I[c] |
| 19 | s | 88 | 377 | 4·3 | >843 | >9·6 | C |
| 20 | s | 89 | 257 | 2·9 | 819 | 9·2 | C |

Duration of the log CEA phase and survival of the patients are explained in Table I.
[a] For abbreviations see Table I.
[b] C = chemotherapy, I = immunotherapy, R = radiotherapy, O = second look surgery, OO = second and third look surgery.
[c] Immunization incomplete.

the standard deviation increased simultaneously which means that the various treatments were not equally effective in individual patients.

*Patients with visceral and other metastases*

Another group of patients with recurrent disease exhibiting exponential increases of circulating CEA during recurrence had developed visceral metastases other than liver metastases (20/87), brain metastases (1/87) or bone metastases (3/87). Selected CEA time courses and their semilogarithmic plots are shown in Fig. 3(a), (b).

Fourteen of these 24 patients received treatment of recurrent disease or underwent second-look surgery. In Table III the CEA doubling times, the therapeutic modalities and survival after the initial CEA rise of the log CEA phase are given for 20 patients. Patients under surveillance for less than 9 CEA doubling times but still alive are not included in Table III. In the 9 patients with untreated visceral metastasis a correlation between CEA doubling time and survival of patients could not be established. Some of the patients fitted the correlation plot given in Fig. 2 for patients with liver metastasis. This might be explained by the possibility that a proportion of patients with visceral metastases also had liver metastases not readily detected by the clinical methods used.

Though the question whether individual CEA doubling times also correlate with survival in the group of patients with untreated visceral metastasis still remained open, it was found that the survival times expressed in multiples of individual CEA doubling times reflected quite accurately the effect of treatment on patient survival. The mean survival after the initial CEA rise of the log CEA phase was $4\cdot3 \pm 2\cdot3$ CEA doubling times for patients without

FIG. 3.—Time course of circulating CEA in patients with resected colorectal carcinoma developing visceral metastasis (× ; ▼) or second primary carcinomas (●; ■): (a) linear plot; (b) semi-log plot.

treatment, whereas the treated patients had a mean survival of $9 \cdot 6 \pm 4 \cdot 2$ CEA doubling times ($P = 0 \cdot 003$). In some cases, the individual survival data indicated that treatment of recurrent disease was not very effective. The CEA doubling times calculated from the CEA time courses of patients who developed bone metastases were relatively short. In the 3 cases registered to date the CEA doubling times were 54, 55 and 60 days. The CEA time course of a patient who developed brain metastasis exhibited a distinctly longer CEA doubling time of 598 days.

*Patients with local recurrence*

To date we have registered 14 cases of local recurrence exhibiting a log CEA phase in the CEA time course. Six patients underwent second-look surgery and 3/6 remained disease-free. All others showed further disease progression and the corresponding CEA time courses exhibited biphasic log CEA slopes. The CEA time courses of 2 selected cases are given in Fig. 4. The calculated individual CEA doubling times and the duration of the log CEA phase of local recurrence are listed together with the site of recurrence and the diagnosis of disease progression (Table IV). The CEA doubling times ranged from 142 to 868 days. Only the CEA doubling time of a developing brain metastasis (598 days) was comparable with the higher CEA doubling times of local recurrence. In this special case local recurrence was definitely excluded by a preceding negative second-look operation.

### DISCUSSION

The data obtained from our retrospective study of the postoperative CEA time courses of colorectal cancer patients with recurrent disease disclosed two relationships. (1) There were phases of exponential CEA increase during recurrent disease in patients with resected colorectal carcinoma. Their establishment essentially depended on the frequency of CEA determinations performed. (2) There was a positive correlation of CEA doubling time and survival for colorectal cancer patients who developed liver metastases without having further treatment.

The question whether the phase of linear relationship between log CEA and time correlates with a corresponding increase of tumour volume in man as was observed in nude mice xenografted with human tumour cells (Raghavan *et al.*, 1980; Staab

TABLE III.—*CEA doubling times of patients who developed metastasis other than liver metastasis after primary resection and, in part, underwent various postoperative treatments of recurrent disease*

| Patients | Primary tumour[a] | CEA doubling time days | Duration of log CEA phase: Days | Duration of log CEA phase: CEA doubling times (multiples) | Survival after initial CEA increase: Days | Survival after initial CEA increase: CEA doubling times (multiples) | Post-operative therapy[a] |
|---|---|---|---|---|---|---|---|
| Visceral met. | | | | | | | |
| 1 | s | 65 | 195 | 3·0 | 330 | 5·1 | — |
| 2 | s | 72 | 167 | 2·3 | 603 | 8·4 | — |
| 3 | s | 99 | 557 | 5·6 | 739 | 7·5 | — |
| 4 | s | 110 | 289 | 2·6 | 408 | 3·7 | — |
| 5 | r | 118 | 84 | 0·7 | 309 | 2·6 | — |
| 6 | s | 123 | 389 | 3·2 | 461 | 3·7 | — |
| 7 | c | 136 | 217 | 1·6 | 367 | 2·7 | — |
| 8 | s | 230 | 261 | 1·1 | 473 | 2·1 | — |
| 9[b] | c | 231 | 479 | 2·1 | 571 | 2·5 | — |
| 10[c] | c | 47 | 465 | 9·9 | 622 | 13·2 | O |
| 11 | s | 50 | 98 | 2·0 | 754 | 15·1 | O |
| 12 | r | 76 | 166 | 2·2 | 975 | 12·8 | C |
| 13 | r | 89 | 227 | 2·6 | >811 | >9·1 | O, R |
| 14 | r | 92 | 114 | 1·2 | 604 | 6·6 | O |
| 15 | r | 129 | 336 | 2·6 | 378 | 2·9 | C |
| 16 | r | 132 | 251 | 1·9 | 1509 | 11·4 | C |
| 17 | r | 150 | 691 | 4·6 | 1301 | 8·7 | O, C |
| 18 | r | 167 | 395 | 2·4 | 1000 | 6·0 | C |
| Brain met. | | | | | | | |
| 1 | s | 598 | 547 | 0·9 | 605 | 1·0 | — |
| Bone met. | | | | | | | |
| 1 | r | 60 | 111 | 1·9 | >666 | >11·1 | R, C |

Duration of the log CEA phase and survival of patients are as in Table I.
[a] For abbreviations see Table I + II.
[b] Visceral and lung metastasis.
[c] Visceral and skin metastasis.

TABLE IV.—*CEA doubling times of patients who developed local recurrences or second primary carcinomas*

| Patients | Primary tumour | Site of recurrence | CEA doubling time days | Duration of log CEA phase days | Disease progression |
|---|---|---|---|---|---|
| 1 | r | Sacral | 142 | 176 | Visc. mets. |
| 2 | r | Sacral | 144 | 225 | Visc. mets. |
| 3 | r | Sec. primary | 168 | 420 | Liver mets. |
| 4 | c | Sec. primary | 221 | 133 | Visc. mets. |
| 5 | r | Sacral | 251 | 156 | Visc. mets. |
| 6 | r | Local | 258 | 274 | Bone mets. |
| 7 | r | Sacral | 264 | 522 | Bone Mets. |
| 8 | s | Sec. primary | 272 | 371 | Disease free[a] |
| 9 | s | Local | 328 | 98 | Liver mets. |
| 10 | r | Sacral | 356 | 414 | Pelvis mets. |
| 11 | r | Sacral | 362 | 363 | Disease free[b] |
| 12 | s | Local | 736 | 190 | Disease free[c] |
| 13 | s | Local | 770 | 282 | Liver mets. |
| 14 | r | Local | 868 | 218 | Bone mets. |

[a] After second-look surgery for > 852 days.
[b] After second-look surgery for > 473 days.
[c] After immunotherapy, second-look surgery and chemotherapy for > 803 days.

FIG. 4.—Biphasic time course of circulating CEA in patients with resected colorectal carcinomas developing local recurrence followed by visceral (●) or liver metastasis (■): a linear plot; (b) semi-log plot.

& Anderer, 1981, 1982) remains presently unanswered. The critical factor is the release of CEA from the tumour cells and the molecular mechanism by which it is governed, since they determine the CEA doubling times during the exponential CEA increase. The fact that during progressive increases of circulating AFP and CEA a linear relationship between log AFP or log CEA and time is observed, implies that in these particular phases the rates of release and degradation of these tumour markers are relatively constant.

In this retrospective study we obtained CEA doubling times in developing liver metastases which ranged from 10 to 102 days. The individual CEA doubling times correlated positively with survival of the patients, calculated from the data of initial log CEA increase, and the untreated patients showed a mean survival of 7·0 individual CEA doubling times $\pm$ 1·8. At the beginning of the exponential increase of circulating CEA and subsequently at the time of diagnosis the patients usually were symptom free, independent of CEA doubling time. Assuming that the CEA increase also correlated with tumour volume in man, survival might be limited by a critical tumour burden leading to secondary complications in the liver. Survival expressed in multiples of the individual CEA doubling times also proved to reflect directly the effect of treatment of liver metastasis on tumour development and survival.

For patients with visceral metastases other than liver metastases and no further treatment, a correlation between CEA doubling time and survival could not be established. This could be due to the limited number of patients in this group or more probably to the possibility that CEA-releasing cells in these metastatic cases were not representative of the exacerbation of malignant disease which might entail tumour cells not releasing CEA. Furthermore, the mean survival of these patients, expressed in multiples of CEA doubling times, were distinctly shorter than for patients developing liver metastasis possibly due to secondary complications leading to serious damage of normal physiological functions earlier during tumour development. In a recent report (Toth *et al.*, 1982) it was demonstrated that CEA is rapidly removed from circulation by the liver and therefore distinct impairment of liver function might influence clearance of CEA giving rise to shorter CEA doubling times.

The question whether the correlation between CEA doubling times and survival in patients with untreated liver metastasis is based on CEA release from proliferating metastatic cells or on failure of CEA clearance by the liver or on both mechanisms cannot be answered presently. Further investigations on the biochemical regulation of CEA release, degradation and clearance are necessary.

The findings obtained in our retrospective study need confirmation by a prospective study randomized with respect to other prognostic criteria including performance status at the start of the exponential CEA increase. However, since CEA doubling times predicted survival in colorectal cancer patients with liver metastasis who were not treated, clinical trials claiming improvements in overall survival should be corrected for any imbalance in distribution of CEA doubling times.

The authors thank Ms S. Glock and Ms S. Kraus for excellent technical assistance.

### REFERENCES

JOHNSON, P. J. & WILLIAMS, R. (1980) Serum alpha-feto-protein estimations and doubling time in hepatocellular carcinoma: influence of therapy and possible value in early detection. *J. Natl Cancer Inst.*, **64**, 1329.

RAGHAVAN, D., GIBBS, J., NOGUEIRA COSTA, R. & 4 others (1980) The interpretation of marker protein assays: a critical appraisal in clinical studies and a xenograft model. *Br. J. Cancer*, **41**, (Suppl. IV), 191.

STAAB, H. J. & ANDERER, F. A. (1981) Correlation of growth of human tumor cell lines with circulating CEA in nude mice. *Oncodevelop. Biol. Med.*, **2**, 24.

STAAB, H. J. & ANDERER, F. A. (1982) Antisera against circulating human tumor-associated antigens prepared with sera of xenografted athymic mice. *Tumor Diagn. Therap.*, **3**, 29.

STAAB, H. J., ANDERER, F. A., STUMPF, E. & FISCHER, R. (1978) Slope analysis of the post-operative CEA time course and its possible application as an aid in diagnosis of disease progression in gastrointestinal carcinoma. *Am. J. Surg.*, **136**, 322.

STAAB, H. J., ANDERER, F. A., STUMPF, E. & FISCHER, R. (1980) Are circulating CEA immune complexes a prognostic marker in patients with carcinoma of the gastrointestinal tract? *Br. J.* Cancer, **42**, 26.

STEELE, G., ZAMCHECK, N., WILSON, R. & 4 others (1980) Results in CEA-initiated second-look surgery for recurrent colorectal cancer. *Am. J. Surg.*, **139**, 544.

TOTH, C. A., THOMAS, P., BROITMAN, S. A. & ZAMCHECK, N. (1982) A new Kupffer cell receptor mediating plasma clearance of carcinoembryonic antigen by the rat. *Biochem. J.*, **204**, 377.

WOOD, C. B., RATCLIFFE, J. G., BURT, R. W., MALCOLM, A. J. H. & BLUMGART, L. H. (1980) The clinical significance of the pattern of elevated serum carcinoembryonic antigen (CEA) levels in recurrent colorectal cancer. *Br. J. Surg.*, **67**, 46.